# Genetic Diversity, Structure and Effective Population Size of Old-Growth vs. Second-Growth Populations of Keystone and Long-Lived Conifer, Eastern White Pine (*Pinus strobus*): Conservation Value and Climate Adaptation Potential

**DOI:** 10.3389/fgene.2021.650299

**Published:** 2021-08-12

**Authors:** Om P. Rajora, John W. R. Zinck

**Affiliations:** Faculty of Forestry and Environmental Management, University of New Brunswick, Fredericton, NB, Canada

**Keywords:** old-growth and second-growth forests, genetic diversity and population structure, effective population size, SNPs in climate-responsive candidate genes, microsatellites, conservation value, climate adaptive potential, climate change

## Abstract

Whether old-growth (OG) forests have higher genetic diversity and effective population size, consequently higher conservation value and climate adaptive potential than second-growth (SG) forests, remain an unresolved issue. We have tested the hypothesis that old-growth forest tree populations have higher genetic diversity, effective population size (*N_E_*), climate adaptive potential and conservation value and lower genetic differentiation than second-growth forest tree populations, employing a keystone and long-lived conifer, eastern white pine (EWP; *Pinus strobus*). Genetic diversity and population structure of old-growth and second-growth populations of eastern white pine (EWP) were examined using microsatellites of the nuclear and chloroplast genomes and single nucleotide polymorphisms (SNPs) in candidate nuclear genes putatively involved in adaptive responses to climate and underlying multilocus genetic architecture of local adaptation to climate in EWP. Old-growth and second-growth EWP populations had statistically similar genetic diversity, inbreeding coefficient and inter-population genetic differentiation based on nuclear microsatellites (nSSRs) and SNPs. However, old-growth populations had significantly higher chloroplast microsatellites (cpSSRs) haploid diversity than second-growth populations. Old-growth EWP populations had significantly higher coalescence-based historical long-term *N_E_* than second-growth EWP populations, but the linkage disequilibrium (LD)-based contemporary *N_E_* estimates were statistically similar between the old-growth and second-growth EWP populations. Analyses of population genetic structure and inter-population genetic relationships revealed some genetic constitution differences between the old-growth and second-growth EWP populations. Overall, our results suggest that old-growth and second-growth EWP populations have similar genetic resource conservation value. Because old-growth and second-growth EWP populations have similar levels of genetic diversity in genes putatively involved in adaptive responses to climate, old-growth, and second-growth populations may have similar adaptive potential under climate change. Our results could potentially be generalized across most of the boreal and temperate conifer forest trees. Our study contributes to address a long-standing issue, advances research field and knowledge about conservation and ecological and climate adaptation of forest trees.

## Introduction

Whether old-growth (OG) forests have higher conservation value and climate adaptation potential and provide better ecosystem functioning, productivity, and resilience than second-growth (SG) forests, remains a long-standing unresolved issue. Biodiversity provides the foundation of ecosystem stability, functioning, and productivity, as well as provisions of ecosystem services (e.g., Worm et al., 2006; review in Tilman et al., 2014). There is also evidence that standing genetic diversity benefits ecosystem functioning and resilience (e.g., Reusch et al., 2005; Roger et al., 2012; Salo and Gustafsson, 2016). Genetic diversity is the foundation of all biodiversity because it provides the raw material for individuals, populations, and species to evolve and adapt especially under abiotic and biotic stress and changing climate and environmental conditions (Rajora, 1999; Fady et al., 2015, 2020; Hoban et al., 2020). As such, individuals and populations that have high genetic diversity should have better chances of persistence and adaptation under climate change conditions. This may be particularly true for genetic diversity in climate-responsive genes. Hence, genetic diversity in genes underlying adaptive responses to climate may indicate the adaptive potential of individuals and populations under climate change. There is empirical evidence that heterozygosity at candidate genes potentially involved in adaptation to local climate was higher than control genes, and allele frequencies at SNPs associated with local adaption could be used as a predictor of climate maladaptation in *Pinus pinaster* (Jaramillo-Correa et al., 2015). Furthermore, genetic diversity provides the basis for conservation of genetic resources within and across species (e.g., Hoban et al., 2020). Therefore, genetic diversity maintenance is essential for the survival, adaptation, and evolution of individuals, populations, and species, and stability, functioning, productivity and resilience of ecosystems, making genetic diversity and its maintenance an important consideration in conservation, adaptation, and ecosystem management.

Because forest trees are normally the keystone species of the forest ecosystems, their genetic diversity has special significance and genetic diversity has been identified as the foundation of forest sustainability (Rajora, 1999; Rajora and Mosseler, 2001a,b; Lefèvre et al., 2014; Fady et al., 2015, 2020). Old-growth, primary, or virgin forests due to their ecological and other characteristics are generally known to be the reservoirs of species biodiversity (Spies and Franklin, 1996; Spies, 2004) and are assumed to have high genetic diversity. Old-growth forest trees constitute gene pools that are the product of various evolutionary processes over a long period encountering environment and climate heterogeneity over the long temporal scale, potentially resulting in survival of individuals that have high genetic diversity (see also Spies and Franklin, 1996). On the other hand, second-growth forests originate after forest harvesting and/or natural and other anthropogenic disturbances. The forest harvesting and renewal practices and natural disturbances can impact demography and several evolutionary processes, such as genetic drift, gene flow, inbreeding, and selection, by affecting local tree density creating bottleneck, fragmentation, and spatial genetic structure, which can adversely affect genetic diversity and differentiation in post-harvest tree populations (e.g., Buchert et al., 1997; Rajora, 1999; Rajora et al., 2000; review in Lefèvre et al., 2014; Wickneswari et al., 2014). Also, old-growth forest has been shown to have high reproductive capacity and fitness (Mosseler et al., 2003), which may be due to its large effective population size. Therefore, old-growth forest tree populations are thought to have higher inherent genetic diversity, effective population size, and evolutionary potential and conservation value than second-growth forest tree populations. However, there is insufficient empirical evidence to support this notion. Also, because virgin old-growth forest tree populations likely represent ancestral gene pools which have persisted through various climate conditions over a long period, they may have higher adaptive potential than second-growth forest tree populations under climate change conditions. However, there is almost nothing known about this aspect.

Despite fundamental and applied significance, only a few studies have directly compared genetic diversity and differentiation between old-growth and second-growth forests. The results reported are mixed with inconsistent patterns. Old-growth populations have been found to have higher genetic diversity than second-growth populations of *Iriartea deltoidea* (Sezen et al., 2005), *Quercus rubra* (Gerwein and Kesseli, 2006), *Picea asperata* (Wang et al., 2010); *Platycladus orientalis* (Zhu and Lou, 2012), and *Vochysia ferruginea* (Davies et al., 2015). Similar levels of genetic diversity for old-growth and second-growth populations have been reported for *Picea mariana* (Rajora and Pluhar, 2003), *Pinus strobus* (Marquardt and Epperson, 2004; Chhatre and Rajora, 2014), *Q. rubra* (Aldrich et al., 2005), *Sequoia sempervirens* (Brinegar, 2011), and *Picea glauca* (Fageria and Rajora, 2014). Also, in some cases, the patterns reported are different between different markers and different studies for the same species. For *Q. rubra*, Gerwein and Kesseli (2006) reported higher nuclear microsatellites (nSSR) genetic diversity in old-growth than second-growth populations but similar chloroplast microsatellite (cpSSR) genetic diversity for OG and SG populations. In contrast, Aldrich et al. (2005) reported similar nSSR genetic diversity for different age classes of this species. Also, no clear patterns of OG vs. SG genetic diversity have emerged in some comparisons made between preharvest and postharvest forest tree populations in studies on genetic effects of forest management practices (review in Wickneswari et al., 2014).

Almost all studies on genetic diversity of OG vs. SG forests are based on nuclear and/or chloroplast microsatellite, AFLP, and ISSR markers. These markers are presumed to be selectively neutral. Climate change has become a prominent evolutionary driving force in natural terrestrial ecosystems, predominantly occupied by forests. The potential of forest trees populations to adapt under climate change depends upon their genetic make-up and standing genetic variation, especially in genes underlying local adaptation and responses to climate change because diversity in these genes will provide the raw material for adaptive responses to selection under climate change. While substantial research is done on genetic architecture of local adaptation in forest trees (e.g., Jaramillo-Correa et al., 2015; Rajora et al., 2016; Lu et al., 2019; review in Balkenhol et al., 2019; Tyrmi et al., 2020), virtually nothing is known about genetic diversity of old-growth vs. second-growth forest tree populations in genes underlying local adaptation and adaptive responses to climate change. If OG populations have higher genetic diversity than SG populations in genes underlying local adaption and adaptive response to climate, OG populations may have higher adaptive potential than SG populations under climate change, thus higher conservation value.

The objective of this study was to examine whether old-growth forest tree populations have higher genetic diversity and effective population size, thus higher conservation value and adaptive potential, than second-growth forest tree populations, employing eastern white pine (EWP; *Pinus strobus*) as a test species. EWP is an ideal candidate for this study as outlined below in Materials and Methods. We have used microsatellite DNA markers of the nuclear and chloroplast genomes, which are presumed to be selectively neutral, and SNPs in candidate nuclear genes putatively involved in adaptive responses to climate and underlying local adaptation to climate in this species. We tested the hypothesis that OG populations have higher genetic diversity and effective population size and lower inter-population genetic differentiation than SG populations, thus potentially higher conservation value and adaptation potential.

## Materials and Methods

### Study Species

Eastern white pine is highly ecologically and economically important and a keystone species of temperate white pine ecosystems. It is a long-lived (up to 450 years) species and has a wide natural range in eastern North America (Wendel and Smith, 1990). Historically, EWP has played a significant role in the economic development of eastern North America. “Before the European settlement, this species was a predominant tree species in forest landscapes of the Great Lakes region of North America (Buchert et al., 1997).” However, its heavy exploitation over 150 years has caused fragmentation and reduction in population numbers and sizes (Buchert, 1994). EWP has been extensively logged in Canada from late 1800s to 1950s to help European settlement (Wilson and Gray, 2001; Carmean, 2012). First the forest was cleared for settlements and then largest, tallest, and straightest EWP trees were selectively harvested for ships and domestic use (Wilson and Gray, 2001; Carmean, 2012). Currently, this species is of conservation concern over its range. Pristine old-growth (150 + years) EWP forests are almost non-existent. A study conducted in 1993 estimated that the forest cover of EWP virgin old-growth forest in Canada was only about 0.5% that of the pre-settlement EWP forest cover (Quinby, 1993). Ontario still has a very few extant OG EWP populations in remote and previously inaccessible northern and northwestern areas (Perera and Baldwin, 1993), which represent most of the remaining undisturbed gene pools of the species. These populations provide excellent opportunities to examine the genetic conservation value of old-growth populations by comparing their genetic diversity, population structure and effective population size with that of SG stands, and also understanding the adaptive potential of OG vs. SG populations under climate change. EWP is expected to expand its range northwards under anticipated climate change conditions.

### Populations and Sampling

**Figure 1:**
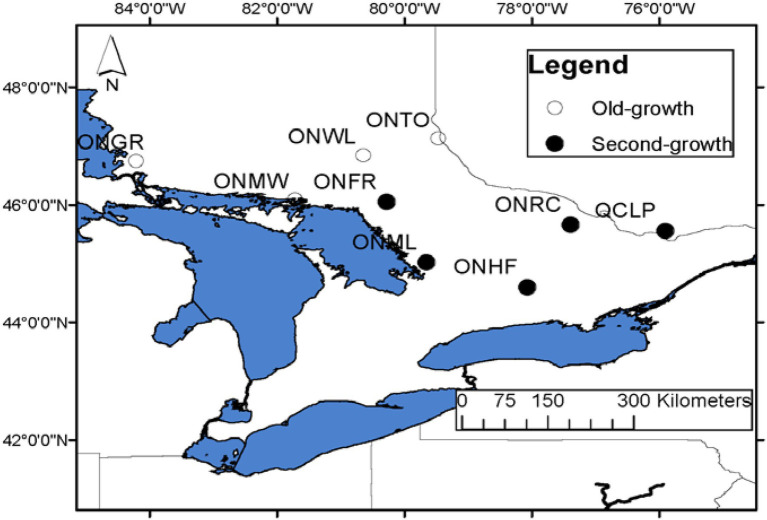
Location map of old-growth (hollow circles) and second-growth (filled circles) EWP populations sampled in eastern Canada. The full names of the populations are provided in Table 1.

We sampled 33 populations throughout the species’ range for several research objectives, including investigating the range-wide population genetic structure and post-glacial phylogeography (Zinck and Rajora, 2016), genetic architecture of local adaption to climate of EWP (Rajora et al., 2016), and old-growth vs. second-growth genetic characteristics. Four of these populations were pristine old-growth located in the natural forest, one population with natural old-growth EWP located in a city park, and the remaining 28 populations were second-growth EWP. All sampled four pristine old-growth populations were located in northern Ontario. These populations represented only a few old-growth EWP populations that exist in Ontario. With the arrival of Europeans, harvesting of EWP started from south and southeastern Ontario to north and northwestern Ontario (Andree Morneault, Silviculture Forester, Nipissing Forest Resource Management Inc., formerly of Ontario Ministry of Natural Resources, personal communication). Thus, a few remnant original old-growth populations could only be found in northern or northwestern Ontario (see also Carmean, 2012), and in remote areas in northwestern Quebec. In this study, we included all four pristine old-growth populations from Ontario and four nearest second-growth EWP populations from Ontario and one from bordering area in Quebec (Figure 1; Table 1). Within the last half century, EWP harvesting practices have changed from selective harvesting of superior trees to seed tree and shelterwood cuts. Most often, natural regeneration after these recent harvesting practices is not adequate because of several factors, especially reduced seed source and widely spaced seed years; thus, the natural regeneration is often supplemented with planted seedlings (Carmean, 2012; Om Rajora, personal observations). In order to avoid sampling of planted trees, we sampled EWP stands, which were at least 60 years old at the time of sampling in 2008. And such populations could not be found in the vicinity of the old-growth populations because those areas were harvested recently in a progressive manner from harvested to unharvested areas. Therefore, it was not possible to sample OG and SG populations that exist at the same location or at a short distance. All nine EWP populations included in this study are from the same central group of the EWP phylogeographic lineage (Zinck and Rajora, 2016); thus, postglacial migration history and geography should not have a significant confounding effect. The estimated age of OG populations ranged from 200 to 230 years, whereas that of SG from 60 to 90 years (Table 1). There was no history of harvesting in the old-growth sampled populations, whereas all five second-growth populations were of post-harvest and/or post-fire origin. We do not have specific history of the sampled second growth populations. However, except for the remote inaccessible areas where extant old-growth EWP populations are located, all EWP in Ontario has gone through selective harvesting (high grading) until 1950s followed later by fire episodes (Wilson and Gray, 2001; Carmean, 2012; Andree Morneault, personal communication). Thus, we think that the second-growth EWP populations we sampled are of post-harvest and/or post-fire origin and they are most likely one or two generations apart from the old-growth populations.

**Table 1:** Eastern white pine (EWP) populations sampled; their geographical coordinates and estimated average age.

Population type	Province	Sample locations	Abbreviated name	Latitude (N)	Longitude (W)	Estimated age (years)
Old growth						
	Ontario	Whitefish reserve	ONMW	46°5'13.02"	81°43'23.63"	200
		Goulais river	ONGR	46°44'57.21"	84°13'19.78"	230
		Wolf lake	ONWL	46°50'35.55"	80°39'10.31"	200
		Timiskaming	ONTO	47°7'50.58"	79°28'42.18"	200
Second growth						
		Bala, Muskoka lake	ONML	45°1'12.60"	79°39'37.01"	70
		French river	ONFR	46°3'8.24"	80°17'3.33"	60
		High falls	ONHF	44°35'50.52"	78°4'47.58"	90
		Renfrew county	ONRC	45°39'49.07"	77°23'46.03"	80
	Quebec	Lac Phillip	QCLP	45°33'44.63"	75°54'34.79"	75

Fifty individual trees were randomly sampled from each population, and needles were collected from individual sampled trees for DNA extraction. A 30 m buffer was left between sampled trees to minimize the chance of sampling siblings. Needles were stored at −20°C in sealed plastic bags, with a 5 g silica desiccant pack, before DNA extraction. DNA from needle samples was extracted and prepared as described in Zinck and Rajora (2016).

### Microsatellite and SNP Genotyping and Data Source

All sampled EWP individuals were genotyped for 12 nuclear microsatellite markers and a subset of 20 individuals per population for three chloroplast microsatellite markers as described in Zinck and Rajora (2016). We obtained the nuclear and chloroplast microsatellite genotypes data for the nine selected eastern white pine populations from Zinck and Rajora (2016).

A subset of 22 randomly selected individuals per population was genotyped for SNPs in candidate genes putatively involved in adaptive responses to climate as described in Rajora et al. (2016). We obtained and used the data for 44 SNPs in 25 candidate genes (Rajora et al., 2016) that met our quality control criteria of call rates greater than 80% and a minor allele frequency greater than 1%. The 25 candidate genes are putatively involved in adaptive responses to cold and drought stresses, photoperiodic response, phenology, growth, development, and maintenance of biological processes, cellular integrity, and functions under stress conditions caused by climate factors (Rajora et al., 2016). Previously, we demonstrated that the multilocus covariances among populations for 44 SNPs in these 25 candidate genes were significantly correlated with climatic variables; thus, primarily reflect adaptation to local climate and environment in EWP (Rajora et al., 2016).

Although we obtained the microsatellite and SNP data from Rajora et al. (2016) and Zinck and Rajora (2016), in the present manuscript, we present and discuss new results on the comparison of genetic diversity, structure, and effective population size of OG versus SG EWP populations and their conservation value and adaptation potential under climate change, which were not addressed in the above publications.

### Data Analysis

#### Genetic Diversity and Effective Population Size

Microchecker (Van Oosterhout et al., 2004) was used to check for null alleles in microsatellite loci and there was no statistically significant evidence for potential presence of null alleles. We tested for non-random association of alleles at different loci, both for microsatellites and SNPs, using linkage disequilibrium (LD) tests in FSTAT (v2.9.4; Goudet, 2003), and Arlequin (v3.5.1.2; Excoffier and Lischer, 2010), respectively. Genetic diversity parameters of individual populations were determined using GenAlEx 6.5 (Peakall and Smouse, 2006). Number of alleles per locus (A) and observed and expected heterozygosity (H_O_ and H_E_) were calculated for the nuclear microsatellite and SNP markers. Number of alleles per locus (A), Shannon’s Information Index (I), haploid diversity (H), and unbiased haploid diversity (uH) were calculated for the chloroplast microsatellite markers. The number of rare alleles (A_Rare_) was determined for nuclear microsatellites at the 1% (allele frequency ≤0.01) and for SNPs at the 5% (allele frequency <0.05) criteria. We also estimated the effective number of alleles (A_E_) per locus and fixation index/inbreeding coefficient (*F* or *F*_IS_) for the nuclear microsatellites and SNPs. We used one way ANOVA to test the statistical significance of differences in genetic diversity parameters and fixation index between old-growth and second-growth population groups. We also tested differences in allelic diversity, heterozygosity, and *F*_IS_ between old-growth and second-growth population groups using the permutation test with 1,000 permutations in FSTAT (v2.9.4; Goudet, 2003).

We estimated both historical long-term and contemporary effective population size (*N_E_*) for each population from the nuclear microsatellite data. The historical long-term *N_E_* was estimated based on the coalescent method using the Markov chain Monte Carlo coalescent genealogy sampler LAMARC (Kuhner, 2006). LAMARC estimates Θ from the genetic data as Θ = 4*N*_E_μ, where μ is the mutation rate. Thus, *N_E_* values could be calculated as Θ/4 μ. We assumed an average nuclear microsatellite mutation rate of 10^−3^ per generation. Thus, *N*_E_ (Coalescent) for each population was calculated as Θ/4*10^−3^. The contemporary *N*_E_ (LD) and its parametric 95% CIs were estimated using the LD method in N_E_Estimator v2 (Do et al., 2014). We used two allele frequency critical values, 0.03 and 0.01, because allele frequencies and sample size affect *N*_E_ estimates from the LD method (Waples and Do, 2010). With increasing critical values, the estimates become conservative, and with low critical value, the estimates are biased upward. Waples and Do (2010) suggested a critical value larger of 0.02 for sample size >25. They found the critical value of 0.03 for a sample size of 100 providing quite an unbiased *N*_E_ estimate. Thus, we used the critical values of 0.03. We also used the critical value of 0.01 because 124 of the 252 alleles, we detected in 12 microsatellite loci had frequency of 0.01 in at least one population. Thus, using the critical value >0.01 removes almost half of the allelic variants from the analysis. While *N*_E_ estimates using the critical value of 0.01 may likely be biased upward, it takes all allelic variation into account in our case. Furthermore, an upward bias should not affect our comparison of *N*_E_ between the old-growth and second-growth EWP populations because the estimates will be biased upward for all populations. The statistical significance of differences in the *N_E_* estimates between the old-growth and second-growth populations was determined using one-way ANOVA.

We asked whether there was any signature of bottleneck in the second-growth populations by using the BOTTLENECK program (Piry et al., 1999). We tested populations under both the infinite allele model (IAM) and two-phase models of evolution. Tests for significance were performed using the Wilcoxon’s test in BOTTLENECK (Piry et al., 1999).

#### Population Genetic Differentiation and Structure

Inter-population genetic differentiation among all, old-growth, and second-growth populations from the nuclear microsatellites and SNPs was determined by using *F*-statistics (Weir and Cockerham, 1984) employing FSTAT (v2.9.4; Goudet, 2003). The 95% CIs for *F*_IS_, *F*_IT_, and *F*_ST_ for all, old-growth, and second-growth populations were determined by bootstrapping (1,000 bootstraps) over the loci using FSTAT separately for two marker sets. The significance of differences in *F*_IS_ and *F*_ST_ between old-growth and second-growth population groups was determined by a permutation test using 1,000 permutations in FSTAT. Hierarchical AMOVA (Excoffier et al., 1992) was also performed to determine the extent of genetic differentiation between old-growth and second-growth population groups, among and within populations using all three data sets separately with 999 permutations using GenAlEx 6.5 (Peakall and Smouse, 2006).

Genetic structure of EWP populations was determined by using a Bayesian model-based genetics approach, STRUCTURE (Pritchard et al., 2000). Multiple runs of STRUCTURE were performed for the nuclear microsatellite and SNP datasets separately, to test *K*-values ranging from 2 to 9, over 20 replications. We used the admixture model and correlated allele frequencies options (Falush et al., 2003), a 100,000 burn-in length and 100,000 MCMC replications for each run. The most likely *K*-value was determined using ΔK parameter of the method of Evanno et al. (2005), using STRUCTURE HARVESTER (Earl and von Holdt, 2012; http://taylor0.biology.ucla.edu/struct_harvest/).

We calculated Nei (1972) genetic distance between populations from all three marker datasets separately. Genetic relationships among populations were visualized by performing principal coordinate (PC) analysis and constructing neighbor joining trees based on these genetic distances. We performed the principal coordinate analysis using GenAlEx 6.5 (Peakall and Smouse, 2006), and the ordination of EWP populations on principal coordinates 1 and 2 was plotted for nuclear microsatellites, nuclear SNPs, and chloroplast microsatellites datasets separately. We constructed neighbor joining (NJ) trees from all three marker data sets separately with 1,000 bootstraps using POPTREE2 (Takezaki et al., 2010). The Newick tree files from POPTREE2 were used in MEGA X (Kumar et al., 2018) to visualize the NJ trees, label the population names, and adjust the quality of the figures.

## Results

### Genetic Diversity and Inbreeding Coefficient

We observed 252 alleles at 12 nuclear microsatellite loci: 200 in the old-growth populations and 213 in the second-growth populations. Of these, 161 were common between the two population types. The OG populations had 39 and SG populations had 52 private alleles. Only two of the OG-specific alleles were present in all four OG populations and absent in the five SG populations. Twenty-three of the 39 private alleles in OG were rare, whereas 14 of the 52 private alleles in SG were rare. We observed 29 chloroplast microsatellite haplotypes: 21 in OG populations and 19 in SG populations; of these, 10 were found only in OG and eight in SG, and 11 were common between OG and SG populations. A total of 88 alleles were observed at 44 SNP loci, 84 in four OG, and 85 in five SG populations. Of these, 81 were common between the OG and SG populations. Three alleles were specific to OG and four to SG. However, there was only one allele that was present in SG populations and not in OG populations. No evidence of linkage disequilibrium was observed.

The genetic diversity levels for the mean number of alleles (A) per locus, effective number of alleles per locus (A_E_), and observed (H_O_) and expected (H_E_) heterozygosity from each of the nuclear microsatellites and SNPs were similar between the old-growth and second-growth populations (Tables 2 and 3). The differences between OG and SG for these genetic diversity parameters were not statistically significant based on both ANOVA and permutation tests (all values of *p* > 0.05). Likewise, the number of alleles per locus for the chloroplast microsatellites was not significantly different between OG and SG (*p* > 0.05). However, Shannon’s Information Index (I), haploid diversity (H), and unbiased haploid diversity (uH) for the chloroplast microsatellites were statistically higher in OG than in SG populations (*p* < 0.05; Table 4). On average, the OG populations had higher number of rare alleles for nuclear microsatellites than the SG populations (Table 2) but the differences were not statistically significant (*p* > 0.05). On average, the SG populations had significantly higher number of rare alleles for SNP markers than the OG populations (Table 3). Inbreeding coefficient calculated as *F* or *F*_IS_ from *F*-statistics showed reversed trends between the nuclear microsatellites and SNPs (Tables 2, 3, and 5). Heterozygote deficiency was observed in seven of the nine populations for nuclear microsatellites. On the other hand, we observed heterozygote excess for SNP markers in eight of the nine populations. On average, SG populations showed higher *F* or *F*_IS_ than the OG populations for nuclear microsatellites (Tables 2 and 5) but the differences were not statistically significant (*p* > 0.05) as demonstrated by ANOVA, permutation test, and bootstrap 95% CIs. In contrast, SG populations showed higher excess of heterozygotes than OG populations for SNP markers (Tables 3 and 5) but again the differences for *F* were not statistically significant between the OG and SG populations (*p* > 0.05). Therefore, overall, the inbreeding coefficients were not significantly different between the OG and SG populations. No instances of significant bottlenecks were identified in any population from the BOTTLENECK test.

**Table 2:** Genetic diversity parameters, fixation index and their (SE), and effective population size and their (95% CIs) for EWP populations based on 12 nuclear microsatellite markers.

Population Type	Population	A	A_E_	A_Rare_	H_O_	H_E_	*F*	*N_E_* (Coalescence)	*N_E_* (LD; 0.01)	*N_E_* (LD; 0.03)
Old growth										
	ONMW	10.67 (1.64)	4.53 (0.79)	41	0.632 (0.058)	0.684 (0.057)	0.075 (0.036)	199	360 (189–2,254)	69 (51–100)
	ONGR	10.17 (0.97)	4.76 (0.61)	33	0.767 (0.041)	0.752 (0.029)	−0.023 (0.050)	322	456 (210-Inf)	121 (78–243)
	ONWL	9.83 (1.76)	4.68 (1.05)	24	0.556 (0.065)	0.651 (0.067)	0.142 (0.043)	174	279 (156–1,024)	157 (93–405)
	ONTO	9.83 (1.30)	5.14 (0.77)	23	0.802 (0.056)	0.752 (0.036)	−0.070 (0.064)	314	655 (239-Inf)	176 (102–508)
Second growth										
	ONML	10.33 (1.57)	5.21 (0.90)	27	0.697 (0.082)	0.712 (0.059)	0.062 (0.060)	174	327 (177–1,596)	188 (108–573)
	ONFR	10.17 (1.64)	5.41 (0.92)	26	0.736 (0.072)	0.735 (0.053)	0.014 (0.058)	131	Infinite (561-Inf)	222 (118–1,066)
	ONHF	10.67 (1.67)	4.69 (0.71)	30	0.619 (0.065)	0.716 (0.048)	0.158 (0.049)	109	136 (99–207)	97 (70–153)
	ONRC	9.17 (0.95)	4.23 (0.42)	25	0.590 (0.063)	0.745 (0.018)	0.212 (0.082)	156	233 (137–674)	88 (60–151)
	QCLP	9.17 (1.09)	4.05 (0.51)	23	0.626 (0.059)	0.702 (0.038)	0.104 (0.073)	150	278 (151–1,235)	203 (105–1,234)
Old-growth mean		10.13 (0.70)	4.78 (0.40)	30.2	0.689 (0.031)	0.710 (0.025)	0.031 (0.027)	252^*^	437 (81)	131 (24)
Second-growth mean		9.90 (0.62)	4.72 (0.32)	26.2	0.654 (0.030)	0.722 (0.020)	0.110 (0.030)	144^*^	243 (41)	159 (28)
Grand mean		10.01 (0.46)	4.74 (0.25)	28.0	0.669 (0.022)	0.717 (0.016)	0.075 (0.021)	192	340 (58)	145 (18)

A, number of alleles per locus; A_E_, effective number of alleles per locus; A_Rare_, total number of rare alleles; H_O_, observed heterozygosity; H_E_, expected heterozygosity; *F*, fixation index; and N_E_ (Coalescence), long-term effective population size based on coalescence genealogy; N_E_ (LD), contemporary effective population size based on linkage disequilibrium (LD). The N_E_ means followed by ^*^ are significantly different (*p* < 0.05) between old-growth and second-growth populations. See Table 1 for population information.

**Table 3:** Genetic diversity parameters, fixation index and their (SE) for EWP populations based on 44 SNP markers.

Population type	Population	P	A	A_E_	A_Rare_	H_O_	H_E_	*F*
Old growth								
	ONMW	58.62	1.57 (0.08)	1.29 (0.06)	3	0.239 (0.046)	0.173 (0.029)	−0.271 (0.050)
	ONGR	56.82	1.57 (0.07)	1.25 (0.05)	6	0.165 (0.040)	0.153 (0.027)	−0.038 (0.079)
	ONWL	63.64	1.64 (0.07)	1.29 (0.05)	5	0.217 (0.039)	0.178 (0.028)	−0.133 (0.052)
	ONTO	77.27	1.77 (0.06)	1.34 (0.05)	7	0.213 (0.04)	0.208 (0.03)	0.047 (0.070)
Second growth								
	ONML	72.73	1.73 (0.07)	1.30 (0.05)	8	0.227 (0.038)	0.186 (0.027)	−0.124 (0.049)
	ONFR	75.00	1.75 (0.07)	1.39 (0.06)	8	0.306 (0.049)	0.229 (0.030)	−0.185 (0.069)
	ONHF	45.45	1.46 (0.08)	1.18 (0.05)	9	0.159 (0.046)	0.104 (0.026)	−0.238 (0.069)
	ONRC	63.64	1.64 (0.07)	1.30 (0.05)	6	0.236 (0.042)	0.183 (0.029)	−0.187 (0.054)
	QCLP	77.27	1.77 (0.06)	1.34 (0.05)	7	0.231 (0.036)	0.212 (0.027)	−0.043 (0.059)
Old-growth mean		63.64 (4.82)	1.64 (0.04)	1.29 (0.03)	5.3^*^	0.209 (0.020)	0.178 (0.014)	−0.088 (0.033)
Second-growth mean		66.82 (5.82)	1.67 (0.03)	1.30 (0.02)	7.6^*^	0.232 (0.019)	0.183 (0.013)	−0.146 (0.027)
Grand mean		65.40 (3.69)	1.65 (0.02)	1.30 (0.02)	6.6	0.222 (0.014)	0.181 (0.010)	−0.121 (0.021)

P, percentage of loci polymorphic; A, number of alleles per locus; A_E_, effective number of alleles per locus; A_Rare_, total number or rare alleles; H_O_, observed heterozygosity; H_E_, expected heterozygosity; and *F*, fixation index. A_Rare_ means followed by ^*^ are significantly different (*p* < 0.05) between old-growth and second-growth populations. See Table 1 for population information.

**Table 4:** Chloroplast microsatellite (cpSSR) genetic diversity parameters and their (SE) for EWP populations.

Population type	Population	A	I	H	uH
Old growth					
	ONMW	3.67 (0.33)	0.90 (0.14)	0.48 (0.09)	0.51 (0.09)
	ONGR	3.67 (0.67)	0.94 (0.10)	0.52 (0.06)	0.55 (0.07)
	ONWL	3.67 (0.33)	0.94 (0.11)	0.50 (0.06)	0.53 (0.07)
	ONTO	3.33 (0.33)	0.93 (0.19)	0.53 (0.09)	0.56 (0.09)
Second growth					
	ONML	3.33 (0.33)	0.76 (0.12)	0.42 (0.08)	0.44 (0.08)
	ONFR	3.33 (0.33)	0.89 (0.19)	0.50 (0.12)	0.52 (0.13)
	ONHF	3.33 (0.33)	0.85 (0.13)	0.48 (0.07)	0.50 (0.08)
	ONRC	3.67 (0.33)	0.83 (0.17)	0.45 (0.10)	0.47 (0.11)
	QCLP	2.33 (0.33)	0.63 (0.13)	0.39 (0.07)	0.41 (0.07)
Old-growth mean		3.59 (0.19)	0.93^*^ (0.06)	0.51^*^ (0.03)	0.54^*^ (0.04)
Second-growth mean		3.20 (0.18)	0.79^*^ (0.06)	0.44^*^ (0.04)	0.47^*^ (0.04)
Overall mean		3.37 (0.13)	0.85 (0.04)	0.47 (0.03)	0.50 (0.03)

A, number of alleles per locus; I, Shannon’s Information Index; H, haploid diversity; uH, unbiased haploid diversity. The I, H, and uH means followed by ^*^ are significantly different (*p* < 0.05) between old-growth and second-growth populations. See Table 1 for population information.

**Table 5:** Estimates of Weir and Cockerham (1984) *F*-statistics parameters and their 95% bootstrap CIs (in parenthesis) for EWP populations.

	Genetic marker	*F*_IS_	*F*_IT_	*F*_ST_
All populations				
	Nuclear microsatellites	0.076 (0.023–0.125)	0.160 (0.093–0.224)	0.092 (0.059–0.127)
	Nuclear SNPs	−0.201 (−0.329–−0.054)	−0.120 (−0.258–0.033)	0.067 (0.047–0.092)
Old growth				
	Nuclear microsatellites	0.039 (−0.029–0.113)	0.110 (0.025–0.199)	0.074 (0.039–0.114)
	Nuclear SNPs	−0.150 (−0.304–0.034)	−0.089 (−0.257–0.110)	0.053 (0.027–0.093)
Second growth				
	Nuclear microsatellites	0.105 (0.036–0.170)	0.186 (0.108–0.257)	0.091 (0.060–0.128)
	Nuclear SNPs	−0.239 (−0.362–−0.106)	−0.148 (−0.290–−0.011)	0.074 (0.050–0.100)

### Effective Population Size

The estimated historical long-term *N_E_* (Coalescence) ranged from 109 to 322, with an average of 192 over the nine populations (Table 2). Overall, the OG populations had significantly higher *N_E_* (Coalescence) than the SG populations of EWP (Table 2). The contemporary *N_E_* (LD) at the critical value of 0.01 ranged from 136 to 655, with an average of 340 over eight populations (Table 2). *N_E_* (LD) for the Ontario French River (ONFR) population was estimated to be infinite; thus, this population could not be included in calculating means and significance of differences between the OG and SG populations. In the LD *N_E_* method, infinite estimate is obtained when, by chance, the actual sampling error is larger than the expected, resulting in negative estimate of *N_E_* (Do et al., 2014). In such cases, *N_E_* estimate is interpreted as infinite (Do et al., 2014). On average, OG populations had higher *N_E_* (LD) than SG populations at 0.01 critical value, but the differences were not statistically significant (*p* > 0.05). *N_E_* (LD) estimates at the critical value of 0.03 ranged from 69 to 222, with an average of 145 over the nine populations (Table 2). On average, SG populations had slightly higher *N_E_* (LD) than OG populations, but the differences were not statistically significant (*p* > 0.05).

### Genetic Differentiation

The pair-wise inter-population *F*_ST_ estimates based on microsatellite and SNP markers are provided in Supplementary Tables S1, S2. The mean estimates of Weir and Cochran (1984) *F*-statistics parameters for all, OG and SG populations based on nuclear microsatellites and SNPs are presented in Table 5. Similar levels of inter-population genetic differentiation were observed for OG and SG populations from both nuclear markers, with 5.3% (OG) and 7.4% (SG) inter-population differentiation from SNPs and 7.4% (OG) and 9.1% (SG) from nuclear microsatellites (Table 5). Inter-population genetic differentiation from the *F*_ST_ estimates for all populations was 9.2% for nuclear microsatellites and 6.7% for SNPs. The permutation test and the bootstrap 95% CIs for *F*_ST_ (Table 5) showed that the inter-population genetic differentiation among SG populations was not significantly different from that among OG populations (permutation test two-sided *p* = 0.49 for microsatellites and 0.91 for SNP markers). Hierarchical AMOVA results were consistent with the *F*_ST_ results for inter-population genetic differentiation (Table 6). Only 3% genetic differentiation was observed between OG and SG populations from the nuclear microsatellites and 1% differentiation from each of SNP and chloroplast microsatellite markers. The extent of among-population genetic variation observed was 9, 11, and 0% for nuclear microsatellite, SNP, and chloroplast microsatellite markers, respectively. Overall, both the *F*_ST_ and AMOVA results indicate that about 90% of genetic variation was among individuals within populations.

**Table 6:** Hierarchical portioning of genetic variation from AMOVA.

Source of variation	Percent variance		
	Nuclear microsatellites	Nuclear SNPs	Chloroplast microsatellites
Between OG and SG	3	1	1
Among populations	9	11	0
Among individuals	88	88	99

OG, old-growth; SG, second-growth.

### Genetic Structure

**Figure 2:**
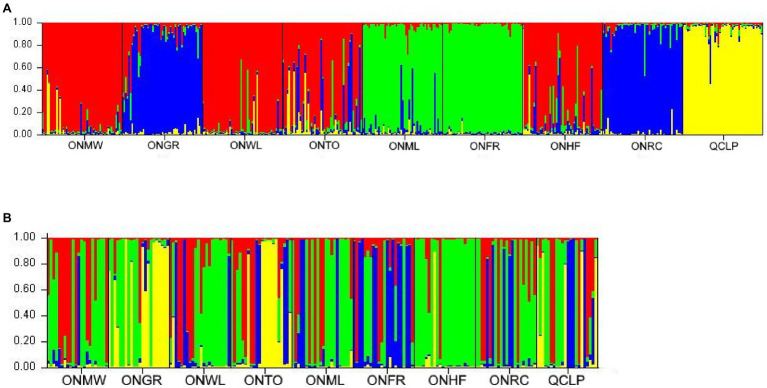
Summary bar plot of estimated membership coefficient (Q) of the studied individuals from nine EWP populations from STRUCTURE analysis from **(A)** nuclear microsatellite markers (four genetic groups, *K* = 4) and **(B)** nuclear single nucleotide polymorphisms (SNPs; four genetic groups, *K* = 4). Each color represents a possible genetic group assignment and each bar represents a single individual. The full names of the populations are provided in Table 1. The plots showing the best *K* value from the nuclear microsatellites and nuclear SNP markers based on the method of Evanno et al. (2005) are in the Supplementary Figure S1.

After performing Evanno et al. (2005) adjustments in STRUCTURE HARVESTER (Earl and von Holdt, 2012), we observed the most prominent peak at *K* = 4 from both nuclear microsatellites and SNPs (Supplementary Figure S1). Thus, STRUCTURE revealed four genetic groups among individuals from each of the two marker sets in the nine EWP populations studied (Figures 2A,B). Based on the similarities of estimated membership coefficient (Q) of the sampled individuals from microsatellite markers, nine populations could be grouped in the following four clusters: 1-ONMW, ONWL, ONTO, and ONHF, 2-ONGR and ONRC, 3-ONML and ONFR, and 4-QCLP (Figure 2A). Thus, three OG populations grouped into the same cluster 1, and the Quebec population was separated from all other populations. As the westernmost Ontario population, ONGR clustered together with the Ontario’s easternmost population ONRC, and geographically closest ONWL and ONFR populations did not belong to the same cluster, the STRUCTURE analysis apparently did not reflect geographical structure of the sampled populations. Based on the similarities of estimated membership coefficient (Q) of the sampled individuals from SNP markers, nine populations could be grouped in the following four clusters: 1-ONMW and ONWL, 2-ONGR and ONTO, 3-ONML, ONFR, ONRC, and QCLP, and 4-ONHF (Figure 2B). Thus, the STRUCTURE analysis from SNP markers separated four SG populations from the four OG populations. The Ontario population ONHF was distinct from the others. Again, the SNP-based population structure was not geographically related. Overall, STRUCTURE results were quite similar between nuclear microsatellites and SNPs.

**Figure 3:**
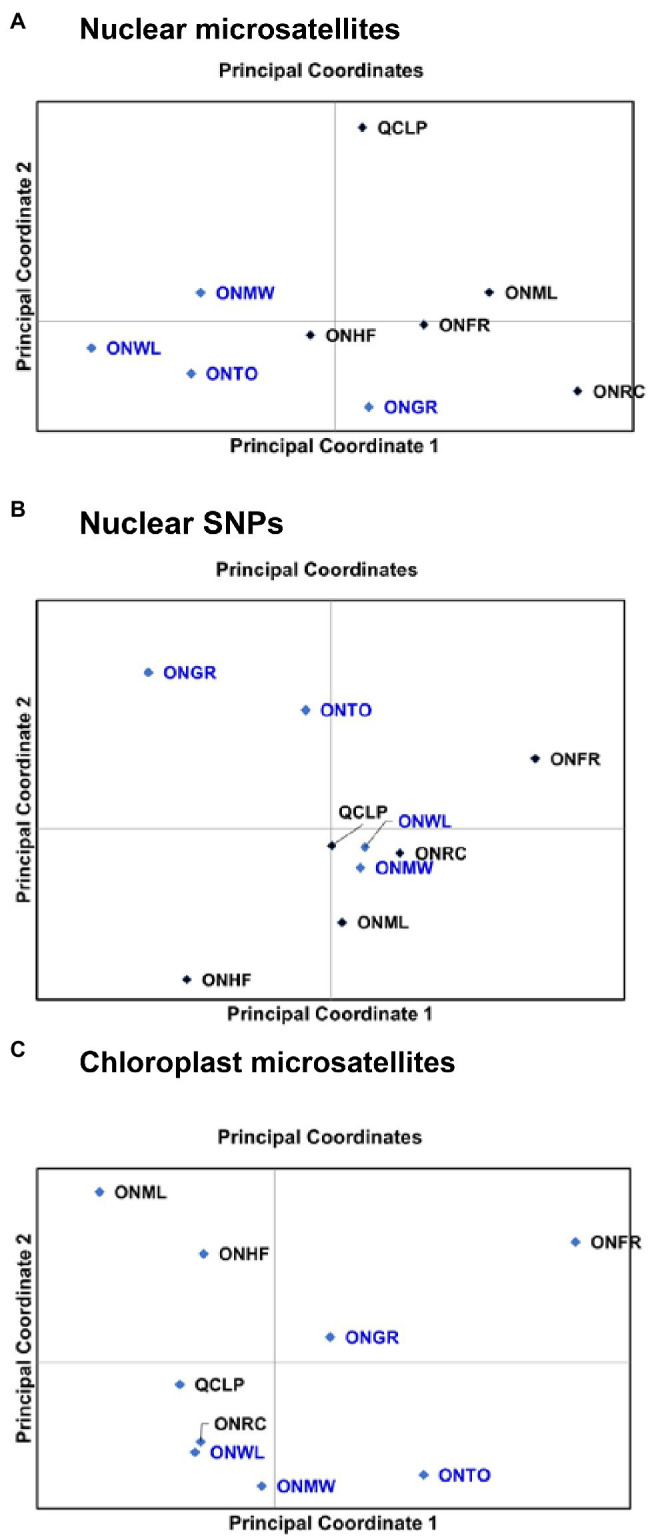
Ordination of nine EWP populations on principal coordinates 1 and 2 from the principal coordinate (PC) analysis based on their genetic distances from **(A)** nuclear microsatellite markers, **(B)** nuclear SNPs, and **(C)** chloroplast microsatellite (cSSR) markers. Details of the populations are provided in Table 1.

The inter-population relationships observed from the principal coordinate (PC) analysis (Figure 3) and NJ trees (Supplementary Figure S2) were similar to those revealed by the STRUCTURE analysis (Figure 2). For nuclear microsatellites, the PC1 (accounting for 28.2% variation) separated ONMW, ONWL, ONTO, and ONHF populations from the other populations, and the PC2 (accounting for 21.5% variation) separated ONWL, ONTO, ONHF, ONGR, ONFR, and ONRC populations from ONMW, QCLP, and ONML populations (Figure 3A). Despite ONML and ONFR populations were separated by the PC2 axis, they clustered closer than any other two population, and the QCLP population clustered separately. Thus, the inter-population genetic relationships from the principal coordinate analysis were highly consistent with that revealed by STRUCTURE. For nuclear SNPs, the PC1 (accounting for 64.7% variation) separated ONGR, ONTO, and ONHF populations from the other populations, and the PC2 (accounting for 16.4% variation) separated ONHF, ONML, ONMW, ONRC, ONWL, and QCLP from ONGR, ONTO, and ONFR populations (Figure 3B). For the chloroplast microsatellites, the PC1 (accounting for 77.8% variation) separated four SG populations ONML, ONHF, QCLP, and ONRC, and two OG populations ONWL and ONMW from the other populations, and the PC2 (accounting for 22.2% variation) separated three OG populations ONMW, ONWL, and ONTO and two SG populations QCLP and ONRC from one OG population ONGR and three SG populations ONHF, ONFR, and ONML (Figure 3C). The genetic relationships and clustering of nine populations from the NJ trees (Supplementary Figure S2) were consistent with the ordination of the populations on principal coordinate 1 and 2 axes (Figure 3), for each of nuclear microsatellites, SNPs and chloroplast microsatellites. Overall, both the principal coordinate and NJ tree analyses show separation between some OG and SG populations.

## Discussion

We have demonstrated that genetic diversity for nuclear microsatellites and SNPs in candidate genes putatively involved in adaptive responses to climate is similar between old-growth and second-growth EWP populations, but OG populations have higher chloroplast microsatellite diversity than SG populations. We have also shown that OG populations on average have higher historical coalescence effective population size than SG populations, but OG and SG eastern white population have statistically similar contemporary effective population size.

### Genetic Diversity, and Inbreeding Coefficient

Genetic diversity for nuclear microsatellites was substantially higher than that for nuclear SNPs in putatively climate-responsive candidate genes. This is expected because microsatellites have higher mutation rates than biallelic SNPs located in functional genes (Hamblin et al., 2007). Irrespective of the differences in the magnitude of genetic diversity levels between nuclear microsatellites and SNPs, our results demonstrate similar patterns of no significant differences in nuclear genetic diversity levels between the studied old-growth and second-growth EWP populations. In contrast, our results demonstrate that the studied old-growth EWP populations have higher chloroplast haploid microsatellite diversity than the second-growth populations, which is in agreement with the general belief and some empirical evidence (Sezen et al., 2005; Gerwein and Kesseli, 2006; Wang et al., 2010; Zhu and Lou, 2012; Davies et al., 2015) that OG forest tree populations have higher genetic diversity than SG populations. All of the studied EWP populations were from the same central phylogeographic lineage and the STRUCTURE analysis did not reveal strict geographical structure among the studied populations, which suggests that postglacial lineage and geography did not have significant confounding effects on these results. However, the nuclear genetic diversity results do not support the notion that OG populations have higher genetic diversity than SG populations and are at somewhat odds with the expectation because eastern white pine populations have been harvested extensively, which has potentially caused fragmentation and bottleneck, the factors that may have reduced genetic diversity in the second-growth populations. The bottleneck test did not show any significant signatures of bottleneck. However, this test detects severe bottleneck over the past 2*N*_E_–4*N*_E_ generations (Piry et al., 1999). Eastern white pine populations have been harvested in the past for about 150–200 years, which represents only a few generations of this species. Thus, this test may not have detected this harvesting bottleneck.

There is evidence that the current harvesting practices can significantly reduce genetic diversity in EWP. Removal of 75% trees (close to seed tree harvesting) in two pristine old-growth EWP stands in northern Ontario significantly reduced genetic diversity (loss of about 25% alleles and about 40–50% of latent genetic potential) in the post-harvest residual gene pool (Buchert et al., 1997; Rajora et al., 2000). Also, shelterwood harvesting involving removal of 20 and 23% of EWP trees in two SG stands in northern Ontario reduced allelic diversity by about 10% and latent genetic potential by about 15% (Rajora, unpublished). Thus, such harvesting practices used for EWP have potential to adversely affect genetic diversity. However, based on the harvesting history of EWP, the parental populations of the studied SG populations in the present study were most likely harvested by high grading (selective harvesting of superior trees), which may have not caused a severe bottleneck in the parental populations. Also, as per the general harvesting and fire history of the study area, the post-harvest residual populations likely experienced fire episodes, which may have benefitted natural regeneration of EWP. These factors may have contributed to the maintenance of genetic diversity in the studied SG EWP populations.

Several biological and other characteristics of EWP may also explain similar nuclear genetic diversity between OG and SG EWP populations. EWP has predominantly outcrossing mating system (*t*_m,_ multilocus outcrossing rates >90%; Rajora et al., 2002) and strong inbreeding depression (Kriebel, 1974). Selection against inbreds can occur at a very early stage even before seed formation in conifers (e.g., O’Connell et al., 2007). Inbreeding and self-fertilization in conifers adversely affect embryo development (Bingham and Squillace, 1955; Franklin, 1970). In the sister species, western white pine (*Pinus monticola*), 80% of the empty seeds could be attributed to inbreeding (Bingham and Squillace, 1955). The studied SG EWP populations were 60–90 years old. It appears that this provided long-enough time for selection against inbreds in the studied SG populations. EWP has long-distance gene flow with highly dispersed pollen (Myers et al., 2007), which could buffer the negative genetic effects of fragmentation from harvesting. Also, EWP can live for over 400 years and has an average lifespan of 250, and the rotation age is 80–100 years (Wendel and Smith, 1990). Thus, only a few generations separate extant trees in second-growth forests from pre-impact virgin old-growth generations because heavy exploitation of EWP started over the past about 150 years (Buchert, 1994; Wilson and Gray, 2001; Carmean, 2012). As stated earlier, the sampled SG populations are most likely one and potentially two generations apart from the sampled OG populations. All of these factors may have prevented the erosion of nuclear genetic diversity in the post-harvest second-growth populations of EWP. The OG-SG EWP genetic diversity results from the nuclear microsatellites and SNPs are consistent with similar results reported earlier in smaller studies (one or two populations comparison) using the same microsatellites (Marquardt and Epperson, 2004; Chhatre and Rajora, 2014). The results are also consistent with no significant differences in genetic diversity levels between old-growth and post-harvest naturally regenerated populations of sympatric conifers, black spruce (*P. mariana*; Rajora and Pluhar, 2003), and white spruce (*P. glauca*; Rajora, 1999; Fageria and Rajora, 2013, 2014).

The chloroplast genome is predominantly uniparentally inherited and virtually has no recombination, with some exceptions (e.g., Rajora and Dancik, 1995). It is paternally inherited in *Pinus* (e.g., Wagner et al., 1987), thus dispersed first *via* pollen and then *via* seed. And gene dispersal *via* pollen occurs over long-distances as compared to that *via* seeds in conifers, including *Pinus*. The studied old-growth EWP populations likely represent the ancestral virgin gene pool, which was likely highly connected throughout its range before extensive harvesting and mortality due to white pine blister (*Cronartium ribicola*) created fragmentations. Thus, the chloroplast genetic diversity in the studied OG populations may represent the pre-harvest ancestral chloroplast genetic diversity. Although, the bottleneck test based on nuclear microsatellites did not detect any significant bottleneck in the studied EWP populations, but this test may not be sensitive enough to detect bottleneck events in the studied populations as discussed above. Harvesting may have caused bottleneck enough for lowering chloroplast genetic diversity in the SG populations, which may not have been buffered by a lack of recombination in the chloroplast genome. The differences in chloroplast genetic diversity between the OG and SG populations were unlikely due to differences in post-glacial migration and evolution because both the OG and SG populations were from the same central lineage group inferred to have originated from the same post-glacial migration route (Zinck and Rajora, 2016).

Despite the different patterns for the magnitude and direction of inbreeding coefficients between microsatellite and SNP markers, our results suggest that the studied OG and SG EWP populations have similar inbreeding rates. This may be due to extensive long-distance gene flow in EWP, which can maintain EWP mating system in a fragmented landscape (e.g., O’Connell et al., 2006).

It is worth noting that genetic diversity levels from all three marker sets and inbreeding levels from nuclear microsatellite and SNP markers of the five second-growth EWP populations studied here are similar to the genetic diversity and inbreeding levels of three (SNPs) or four (nuclear and chloroplast microsatellites) other second-growth EWP populations from Quebec belonging to the species’ same central phylogeographic lineage (Supplementary Tables S3–S5). Also, the *N*_E_ estimates were similar between the five SG EWP populations included here and four other Quebec SG EWP populations (Supplementary Table S3). Therefore, the results of our study could be generalized over a wider range of EWP than we covered in this study.

### Effective Population Size

The results of our study suggest that the old-growth EWP populations have higher historical long-term *N_E_* than the second-growth populations, but old-growth and second growth EWP populations have similar contemporary *N_E_*. This is a significant finding because *N_E_*, particularly contemporary *N_E_*, has long been recognized to play a significant role in conservation and management of genetic resources (e.g., Franklin, 1980; Reiman and Allendorf, 2001; Hoban et al., 2020) because it is directly related to genetic diversity loss due to genetic drift and inbreeding. Similar contemporary *N_E_* between OG and SG populations is likely because these populations may be one generation apart and the LD method, we used, reveals *N_E_* information primarily in the parental generation (Waples and Do, 2010). Although LD method of *N_E_* estimation was found to have greater precision than other methods for microsatellites, the contemporary *N_E_* estimates reported in our study could be considered as crude estimates. For precise estimates, the number of loci, sample size, and critical allele frequency value need to be optimized using information on empirically accurate *N*_E_ of a population as control (see Waples and Do, 2010). We believe that we provide the first LD-based *N_E_* estimates for EWP populations. *N_E_* estimates depend upon allele frequencies, generation times, and mutation rates among other factors, which differ between species, markers, and loci. And it is very difficult to determine precise generation times and mutation rates. Thus, valid comparisons of *N_E_* estimates could be made between studies and species only if the generation time, mutation rates, markers, and methods used are the same. As such, a valid comparison was possible only for the historical *N_E_* estimates. Our coalescent-based historical *N_E_* estimates are consistent with the coalescent-based historical *N_E_* estimates that we reported earlier for OG and SG central and OG marginal populations of EWP from Ontario using the same microsatellite markers but using the MIGRATE program (Chhatre and Rajora, 2014).

### Genetic Differentiation and Population Structure

Since the old-growth populations represented the intact EWP forest and the second-growth populations were potentially affected by fragmentation caused by harvesting and/or fire, a higher genetic differentiation among SG populations than among OG populations was expected. Although the *F*_ST_ estimates were about 23% (nuclear microsatellites) and 40% (nuclear SNPs) higher among the SG populations than among the OG populations of EWP, the differences were not statistically significant. Therefore, our *F*_ST_ results from both nuclear microsatellites and SNPs (Table 5) suggest statistically similar levels of inter-population genetic differentiation for OG and SG populations. This is likely due to extensive long-distance gene flow in EWP (Myers et al., 2007). Although OG and SG populations harbored several private alleles, our AMOVA results demonstrate low (1–3%) genetic differentiation between OG and SG EWP populations (Table 6). However, Bayesian model based SRUCTURE results from both nuclear microsatellites and SNPs, and the principal coordinate analysis results from all three marker sets suggest some genetic constitution differences between the OG and SG EWP populations (Figures 2, 3). Indeed, SNP-based STRUCTURE analysis separated all four OG populations (grouped in two clusters) from all five SG EWP populations (Figure 2B), and nuclear microsatellite-based STRUCTURE analysis separated three OG populations from four SG populations (Figure 2A). The principal coordinate analysis and NJ trees supported the STRUCTURE results. Although the proportion of genetic variation between OG and SG population groups is low from the AMOVA analysis, our overall genetic structure and inter-population genetic distances results show some genetic constitution differentiation between the OG and SG populations. These differences are unlikely due to geographical locations and post-glacial migration and evolution because all OG and SG populations were from the same central phylogeographic lineage (Zinck and Rajora, 2016), and the studied populations did not cluster strictly along their geographical coordinates. This genetic distinction between OG and SG populations may have been caused by the harvesting practice of high grading used in the parental generation of the SG populations because high grading removed superior (tallest, straightest, largest) trees from the population. Higher genetic differentiation between the extant OG populations (if they are left unharvested) and the next-generation SG populations may occur if EWP does not regenerate well naturally after currently used shelterwood harvesting and the seedlings planted to supplement the natural regeneration are from a different or narrow gene pool.

### Genetic Resource Conservation and Adaptive Potential Under Climate Change

Genetic diversity provides the basis for conservation of genetic resources of a species and its populations. Effective population size is an important parameter of high genetic conservation relevance (see Reiman and Allendorf, 2001; Hoban et al., 2020). Therefore, populations that have high standing genetic diversity and high contemporary *N_E_* have high genetic conservation value. We observed that the OG and SG populations of EWP have similar levels of genetic diversity in nuclear microsatellites and SNPs in climate-responsive genes, inter-population genetic differentiation, and contemporary *N_E_*. Therefore, we can infer that the studied extant OG and SG EWP populations have similar value for genetic resource conservation of the species. Since old-growth stands are considered to have the highest species biodiversity, conservation, and ecosystem functioning value (Spies and Franklin, 1996; Spies, 2004), and many animals, birds and other wildlife species continue to rely on EWP old-growth stands to maintain their habitats (Green, 1992; Perera and Baldwin, 1993; Wilson and Gray, 2001), old-growth EWP populations may have higher overall biodiversity conservation value than SG populations. Therefore, it will be prudent to preserve all remnant scant old-growth EWP populations.

Eastern white pine has experienced multiple episodes of post-glacial range expansion and retraction (Ritchie, 1987), encountering fluctuations in climatic and topographical factors over time and space, and is expected to extend its range northward under anticipated climate change conditions. Earlier, we have shown that multilocus covariance among populations for 44 SNPs in 25 candidate genes, we used in this study was associated with local climatic variables, reflecting adaptive responses to local climate in EWP (Rajora et al., 2016). Le Corre and Kremer (2012) have shown that genetic covariance among large number of genes instead of allele frequency changes at a few selected loci underlie local adaptation in forest trees. As discussed in Rajora et al. (2016), selection pressures to local adaptation in the northern portion of the EWP range are relatively novel and recent; thus, the first responses to diversifying selection in response to climate change should be the build-up of excessive multilocus covariances among populations (see also Le Corre and Kremer, 2003, 2012). Because OG and SG EWP populations had similar levels of genetic diversity in 25 candidate genes putatively involved in adaptive responses to climate, it may be inferred that the OG and SG EWP populations may have similar adaptive genetic potential under climate change conditions.

## Conclusion and Broad Implications

Old-growth and second-growth populations of EWP have similar levels of genetic diversity in nuclear microsatellites and SNPs located in candidate genes putatively involved in adaptive responses to climate, and statistically similar levels of inter-population genetic differentiation. However, OG populations have higher chloroplast microsatellites haploid genetic diversity than SG populations. Old-growth EWP populations have higher coalescence-based historical *N_E_*, but their contemporary *N_E_* is similar to that of second-growth populations. Overall, it could be inferred that the studied OG and SG EWP populations have similar genetic conservation value and adaptive potential under climate change. Nevertheless, there are some genetic constitution differences between the OG and SG EWP populations, which may have been caused by high grading harvesting in the parental stands of the SG populations. As the studied SG populations are most likely one generation apart from the OG populations, significant genetic erosion has not yet occurred in these populations. However, genetic diversity may be reduced in SG populations and differentiation between OG and SG populations may be increased if the extant EWP SG populations are not harvested and managed in a genetically sustainable manner. Because extant OG EWP populations are rare, which may be harvested in near future, and most of the EWP forests are SG, it is essential that SG forests are sustainably managed for conservation of their genetic resources, and measures should be taken so that no genetic erosion happens in future generations.

Our study is based on a limited number of genetic markers, although including both putatively neutral microsatellites and adaptively selective SNPs in climate-responsive candidate genes, genome-wide genetic diversity may provide much more comprehensive and more precise inferences. Also, an ideal sampling design should include adjacent OG and SG stands or preharvest OG and postharvest SG populations from the same stand. However, such design was not possible because appropriate OG and SG EWP stands in the same neighborhood could not be found as discussed in Material and Methods. And a study before and after harvest in the same OG populations will require controlled experiments and decades of waiting for SG populations to attain an appropriate sampling age.

While our study used EWP as a test species, the results have broad implications and significance. EWP is a good representative of most of the temperate and boreal conifer forest trees because of its similar biological traits. Like EWP, most of the boreal and northern temperate conifer forest trees have predominantly outcrossing mating system, severe inbreeding depression, selection against inbreds at an early developmental stage, extensive and long-distance gene flow *via* pollen and seed dispersal, paternal transmission of the chloroplast genome, and long generation life cycle. Therefore, the results from our study could possibly be generalized for most of the boreal and northern temperate conifer and potentially to other forest trees. Boreal and temperate forest biomes are the largest terrestrial biomes on earth. Forests cover about one-third of the earth’s land surface, about 33% of which is covered by the boreal zone and about 25% by the temperate zone. Thus, boreal and temperate forest biomes cover about 20% of the planet’s terrestrial surface. As such, they have immense environmental, ecological, economic, and social important. Conifers, such as *Pinus* and *Picea* are the dominant components of the boreal forest, which represents the huge carbon sink. Thus, both the boreal and northern temperate conifer forest trees are hugely important for addressing the climate change challenges. Their conservation and sustainable management are important for the sustainability, stability, and functioning of boreal and temperate ecosystems, provision of ecosystem and environmental services, which are vital for the sustenance of human and other life. Also, many of the boreal and temperate forest trees are anticipated to extend their rages northward under climate change. Their potential to adapt to new climate conditions will depend upon their adaptive and evolutionary potential, which could be reflected by genetic diversity in genes involved in adaptive responses to climate. Old-growth forests, due to their existence for long time and surviving through various temporal variations in climate, are expected to have higher adaptive potential under changing climate conditions. However, most of the old-growth forests have been replaced by second-growth forests, and whether the adaptive potential of these forests is lower than primary old-growth forests is a question of broad implications. Our study addresses this very basic and applied question. Thus, overall, our study should have very wide implications for the conservation and ecological adaptation of forest trees, especially under climate change and the advancement of research in these areas.

## Data Availability Statement

Publicly available datasets, produced earlier by the authors of this article, were analyzed in this study. This data can be found at: https://bmcevolbiol.biomedcentral.com/track/pdf/10.1186/s12862-016-0624-1 and https://journals.plos.org/plosone/article/file?id=10.1371/journal.pone.0158691&type=printable.

## Author Contributions

OR conceived, supervised, and directed the study, obtained the funding, reanalyzed the data, and rewrote and revised the manuscript. JZ conducted the field sampling and all lab work, produced the genotype data, performed initial data analysis, and prepared the initial draft of the manuscript. All authors contributed to the article and approved the submitted version.

## Conflict of Interest

The authors declare that the research was conducted in the absence of any commercial or financial relationships that could be construed as a potential conflict of interest.

## Publisher’s Note

All claims expressed in this article are solely those of the authors and do not necessarily represent those of their affiliated organizations, or those of the publisher, the editors and the reviewers. Any product that may be evaluated in this article, or claim that may be made by its manufacturer, is not guaranteed or endorsed by the publisher.

## Supplementary Material

Supplementary Figure S1 Summary scatterplot of Delta *K* values for eastern white pine populations testing (*K*=) 2–9 clusters, calculated from the STRUCTURE results using the Evanno et al. (2005) method in Structure Harvester. **(A)** Nuclear microsatellites; and **(B)** nuclear SNPs.

Supplementary Figure S2 Neighbor joining tree of eastern white pine populations based on Nei’s (1972) genetic distances constructed using **(A**) nuclear microsatellite markers, **(B)** nuclear SNP markers, and **(C)** chloroplast microsatellite markers. The number on the nodes represent the percent bootstrap support from 1,000 bootstraps. Details of the populations are provided in Table 1.

Supplementary Table S1 Pairwise *F*_ST_ estimates between eastern white pine populations based on nuclear microsatellites.

Supplementary Table S2 Pairwise *F*_ST_ estimates between eastern white pine populations based on SNP markers.

Supplementary Table S3 Genetic diversity parameters, fixation index and their (SE), and effective population size and their (95% CIs) for four eastern white pine second-growth populations from Quebec based on 12 nuclear microsatellite markers.

Supplementary Table S4 Chloroplast microsatellite genetic diversity parameters and their (SE) for four eastern white pine second-growth populations from Quebec.

Supplementary Table S5 Genetic diversity parameters, fixation index, and their (SE) for three eastern white pine second-growth populations from Quebec based on 44 SNP markers.
