# Supplementary material for: Genetic Diversity, Structure and Effective Population Size of Old-Growth vs. Second-Growth Populations of Keystone and Long-Lived Conifer, Eastern White Pine (Pinus strobus): Conservation Value and Climate Adaptation Potential
Source: Front Genet. 2021 Aug 12;12:650299. doi: 10.3389/fgene.2021.650299 (PMC8388927; doi:10.3389/fgene.2021.650299)
Supplement: Supplementary Figure S1 — Summary scatterplot of Delta K values for eastern white pine populations testing (K=) 2–9 clusters, calculated from the STRUCTURE results using the Evanno et al. (2005) method in Structure Harvester. (A) Nuclear microsatellites; and (B) nuclear SNPs. [file Image_1.pdf]

A

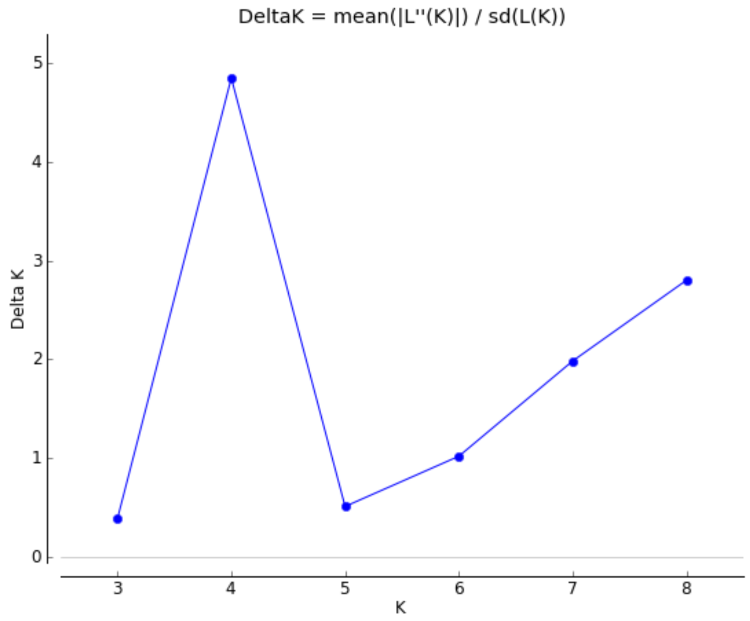

B

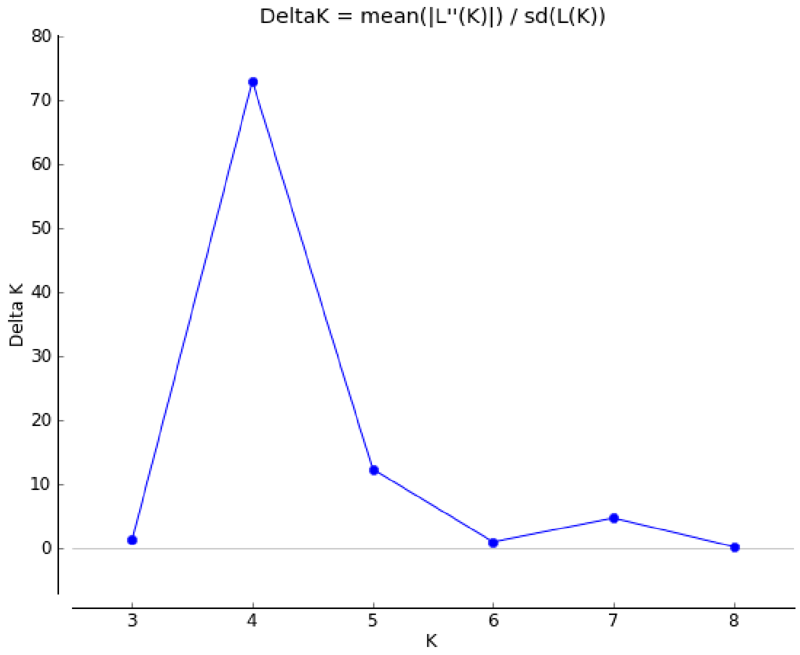

Figure S1. Summary scatterplot of Delta *K* values for eastern white pine populations testing (*K*=) 2 – 9 clusters, calculated from the STRUCTURE results using the Evanno *et al.* (2005) method in Structure Harvester. **(A)** nuclear microsatellites; and **(B)** nuclear SNPs.
