# Supplementary material for: Genetic Diversity, Structure and Effective Population Size of Old-Growth vs. Second-Growth Populations of Keystone and Long-Lived Conifer, Eastern White Pine (Pinus strobus): Conservation Value and Climate Adaptation Potential
Source: Front Genet. 2021 Aug 12;12:650299. doi: 10.3389/fgene.2021.650299 (PMC8388927; doi:10.3389/fgene.2021.650299)
Supplement: Supplementary Figure S2 — Neighbor joining tree of eastern white pine populations based on Nei’s (1972) genetic distances constructed using (A) nuclear microsatellite markers, (B) nuclear SNP markers, and (C) chloroplast microsatellite markers. The number on the nodes represent the percent bootstrap support from 1,000 bootstraps. Details of the populations are provided in Table 1. [file Image_2.pdf]

A Nuclear microsatellites

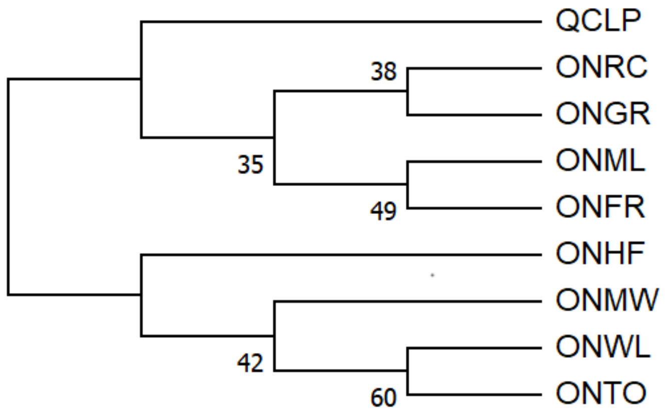

B Nuclear SNPs

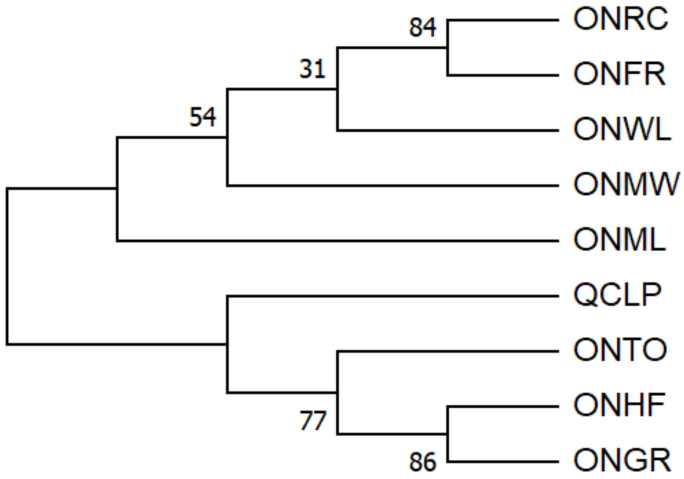

C Chloroplast microsatellites

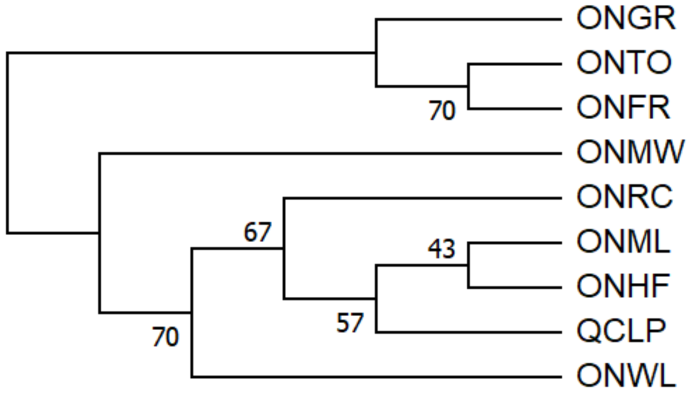

Figure S2. Neighbour joining tree of eastern white pine populations based on Nei's (1972) genetic distances constructed using **(A)** nuclear microsatellite markers, **(B)** nuclear SNP markers, and **(C)** chloroplast microsatellite markers. The number on the nodes represent the percent bootstrap support from 1,000 bootstraps. Details of the populations are provided in **Table 1**.
