# Supplementary material for: Genetic Diversity, Structure and Effective Population Size of Old-Growth vs. Second-Growth Populations of Keystone and Long-Lived Conifer, Eastern White Pine (Pinus strobus): Conservation Value and Climate Adaptation Potential
Source: Front Genet. 2021 Aug 12;12:650299. doi: 10.3389/fgene.2021.650299 (PMC8388927; doi:10.3389/fgene.2021.650299)
Supplement: Supplementary Table S1 — Pairwise FST estimates between eastern white pine populations based on nuclear microsatellites. [file Table_1.pdf]

Table S1. Pairwise F_{ST} estimates between eastern white pine populations based on nuclear microsatellites.

	ONMW	ONGR	ONWL	ONTO	ONML	ONFR	ONHF	ONRC	QCLP
**ONMW**	0.000								
**ONGR**	0.052	0.000							
**ONWL**	0.040	0.064	0.000						
**ONTO**	0.034	0.032	0.030	0.000					
**ONML**	0.054	0.054	0.080	0.054	0.000				
**ONFR**	0.050	0.047	0.061	0.044	0.034	0.000			
**ONHF**	0.030	0.033	0.047	0.033	0.032	0.040	0.000		
**ONRC**	0.073	0.044	0.089	0.052	0.054	0.052	0.053	0.000	
**QCLP**	0.066	0.068	0.087	0.063	0.067	0.070	0.064	0.072	0.000
