# Supplementary material for: Genetic Diversity, Structure and Effective Population Size of Old-Growth vs. Second-Growth Populations of Keystone and Long-Lived Conifer, Eastern White Pine (Pinus strobus): Conservation Value and Climate Adaptation Potential
Source: Front Genet. 2021 Aug 12;12:650299. doi: 10.3389/fgene.2021.650299 (PMC8388927; doi:10.3389/fgene.2021.650299)
Supplement: Supplementary Table S2 — Pairwise FST estimates between eastern white pine populations based on SNP markers. [file Table_2.pdf]

Table S2. Pairwise F_{ST} estimates between eastern white pine populations based on nuclear SNPs.

	**ONMW**	**ONGR**	**ONWL**	**ONTO**	**ONML**	**ONFR**	**ONHF**	**ONRC**	**QCLP**
**ONMW**	0.000								
**ONGR**	0.060	0.000							
**ONWL**	0.008	0.056	0.000						
**ONTO**	0.029	0.037	0.020	0.000					
**ONML**	0.018	0.060	0.014	0.026	0.000				
**ONFR**	0.042	0.111	0.036	0.048	0.037	0.000			
**ONHF**	0.051	0.044	0.049	0.055	0.042	0.110	0.000		
**ONRC**	0.012	0.065	0.007	0.025	0.013	0.029	0.054	0.000	
**QCLP**	0.022	0.050	0.017	0.018	0.013	0.036	0.047	0.018	0.000
