# Supplementary material for: Genetic Diversity, Structure and Effective Population Size of Old-Growth vs. Second-Growth Populations of Keystone and Long-Lived Conifer, Eastern White Pine (Pinus strobus): Conservation Value and Climate Adaptation Potential
Source: Front Genet. 2021 Aug 12;12:650299. doi: 10.3389/fgene.2021.650299 (PMC8388927; doi:10.3389/fgene.2021.650299)
Supplement: Supplementary Table S3 — Genetic diversity parameters, fixation index and their (SE), and effective population size and their (95% CIs) for four eastern white pine second-growth populations from Quebec based on 12 nuclear microsatellite markers. [file Table_3.pdf]

Table S3. Genetic diversity parameters, fixation index and their (SE), and effective population size and their (95% CIs) for four eastern white pine second-growth populations from Quebec based on 12 nuclear microsatellite markers.

Population	Population abbreviation	A	A_E	H_O	H_E	*F*	N_E (LD)
Temiscouata	QCTM	10.25 (0.64)	4.81 (0.42)	0.695 (0.056)	0.774 ().020)	0.093 (0.081)	70 (55-95)
Cap Tourmente	QCCT	9.33 (0.79)	4.36 (0.43)	0.620 (0.061)	0.741 (0.029)	0.147 (0.089)	76 (55-115)
Saint Renyold	QCSR	10.25 (1.33)	4.17 (0.69)	0.563 (0.066)	0.697 (0.041)	0.209 (0.070)	147 (88-367)
Saint Stanilis	QCSS	9.83 (1.56)	4.88 (0.98)	0.652 (0.070)	0.692 (0.052)	0.068 (0.063)	142 (89-308)
	Overall mean	9.92 (0.56)	4.56 (0.33)	0.632 (0.031)	0.726 (0.019)	0.129 (0.038)	109 (20.7)

A, number of alleles per locus; A_E, effective number of alleles per locus; H_O, observed heterozygosity; H_E, expected heterozygosity; *F,* fixation index; N_E (LD), contemporary effective population size based on linkage disequilibrium using allele frequency critical value of 0.03.
