# Supplementary material for: Genetic Diversity, Structure and Effective Population Size of Old-Growth vs. Second-Growth Populations of Keystone and Long-Lived Conifer, Eastern White Pine (Pinus strobus): Conservation Value and Climate Adaptation Potential
Source: Front Genet. 2021 Aug 12;12:650299. doi: 10.3389/fgene.2021.650299 (PMC8388927; doi:10.3389/fgene.2021.650299)
Supplement: Supplementary Table S4 — Chloroplast microsatellite genetic diversity parameters and their (SE) for four eastern white pine second-growth populations from Quebec. [file Table_4.pdf]

Table S4. Chloroplast microsatellite genetic diversity parameters and their (SE) for four eastern white pine second-growth populations from Quebec.

**Population**	**Population abbreviation**	**A**	**I**	**H**	**uH**
Temiscouata	QCTM	2.67 (0.33)	0.743 ().080)	0.447 (0.034)	0.470 (0.036)
Cap Tourmente	QCCT	2.67 (0.33)	0.836 (0.082)	0.525 (0.023)	0.553 (0.024)
Saint Renyold	QCSR	3.00 (0.00)	0.629 (0.062)	0.337 ().040)	0.354 (0.043)
Saint Stanilis	QCSS	2.67 (0.33)	0.534 (0.176)	0.305 (0.114)	0.321 (0.120)
	Overall mean	2.75 (0.13)	0.686 (0.058)	0.403 (0.038)	0.425 (0.040)

A, number of alleles per locus; I, Shannon’s Information Index; H, haploid diversity; uH, unbiased haploid diversity.
