# Supplementary material for: Genetic Diversity, Structure and Effective Population Size of Old-Growth vs. Second-Growth Populations of Keystone and Long-Lived Conifer, Eastern White Pine (Pinus strobus): Conservation Value and Climate Adaptation Potential
Source: Front Genet. 2021 Aug 12;12:650299. doi: 10.3389/fgene.2021.650299 (PMC8388927; doi:10.3389/fgene.2021.650299)
Supplement: Supplementary Table S5 — Genetic diversity parameters, fixation index, and their (SE) for three eastern white pine second-growth populations from Quebec based on 44 SNP markers. [file Table_5.pdf]

Table S5. Genetic diversity parameters, fixation index, and their (SE) for three eastern white pine second-growth populations from Quebec based on 44 SNP markers.

Population	Population abbreviation	P	A	A_E	H_o	H_E	*F*
Cap Tourmente	QCCT	61.4	1.61 (0.07)	1.35 (0.05)	0.266 (0.045)	0.206 (0.030)	-0.186 (0.063)
Saint Renyold	QCSR	79.6	1.80 (0.06)	1.33 (0.05)	0.240 (0.040)	0.206 (0.027)	0.002 (0.073)
Saint Stanilis	QCSS	59.1	1.59 (0.07)	1.31 (0.05)	0.243 (0.043)	0.186 (0.029)	-0.213 (0.056)
	Overall mean	66.7 (6.5)	1.67 (0.04)	1.33 (0.03)	0.250 (0.024)	0.199 (0.016)	-0.119 (0.038)

P, percentage of loci polymorphic; A, number of alleles per locus; A_E, effective number of alleles per locus; H_O, observed heterozygosity; H_E, expected heterozygosity; and *F*, fixation index.
